# Risk prediction models for lung cancer in people who have never smoked: a protocol of a systematic review

**DOI:** 10.1186/s41512-024-00166-4

**Published:** 2024-02-13

**Authors:** Alpamys Issanov, Atul Aravindakshan, Lorri Puil, Martin C. Tammemägi, Stephen Lam, Trevor J. B. Dummer

**Affiliations:** 1 School of Population and Public Health, University of British Columbia, Vancouver, BC V6T 1Z3 Canada; 2 Faculty of Applied Health Sciences, Brock University, St. Catharines, ON Canada; 3 BC Cancer, Provincial Health Services Authority, Vancouver, BC Canada; 4 Department of Medicine, University of British Columbia, Vancouver, BC Canada

**Keywords:** Lung cancer, Prediction model, Never smokers, Systematic review

## Abstract

**Background:**

Lung cancer is one of the most commonly diagnosed cancers and the leading cause of cancer-related death worldwide. Although smoking is the primary cause of the cancer, lung cancer is also commonly diagnosed in people who have never smoked. Currently, the proportion of people who have never smoked diagnosed with lung cancer is increasing. Despite this alarming trend, this population is ineligible for lung screening. With the increasing proportion of people who have never smoked among lung cancer cases, there is a pressing need to develop prediction models to identify high-risk people who have never smoked and include them in lung cancer screening programs. Thus, our systematic review is intended to provide a comprehensive summary of the evidence on existing risk prediction models for lung cancer in people who have never smoked.

**Methods:**

Electronic searches will be conducted in MEDLINE (Ovid), Embase (Ovid), Web of Science Core Collection (Clarivate Analytics), Scopus, and Europe PMC and Open-Access Theses and Dissertations databases. Two reviewers will independently perform title and abstract screening, full-text review, and data extraction using the Covidence review platform. Data extraction will be performed based on the Checklist for Critical Appraisal and Data Extraction for Systematic Reviews of Prediction Modeling Studies (CHARMS). The risk of bias will be evaluated independently by two reviewers using the Prediction model Risk-of-Bias Assessment Tool (PROBAST) tool. If a sufficient number of studies are identified to have externally validated the same prediction model, we will combine model performance measures to evaluate the model’s average predictive accuracy (e.g., calibration, discrimination) across diverse settings and populations and explore sources of heterogeneity.

**Discussion:**

The results of the review will identify risk prediction models for lung cancer in people who have never smoked. These will be useful for researchers planning to develop novel prediction models, and for clinical practitioners and policy makers seeking guidance for clinical decision-making and the formulation of future lung cancer screening strategies for people who have never smoked.

**Systematic review registration:**

This protocol has been registered in PROSPERO under the registration number CRD42023483824.

**Supplementary Information:**

The online version contains supplementary material available at 10.1186/s41512-024-00166-4.

## Introduction

Lung cancer is one of the most commonly diagnosed cancers and the leading cause of cancer-related deaths worldwide. In 2020, more than 2.2 million new lung cancer cases and 1.8 million deaths were estimated to occur, accounting for 1 in 10 cancer cases and 1 in 5 cancer deaths (18% of total cancer deaths) [1]. Patients diagnosed at early stages have substantially better survival prognoses compared to patients diagnosed at advanced stages, with a 5-year survival rate approaching 60% when the cancer is localized. However, most lung cancer cases (56% of all lung cancer) are diagnosed in advanced stages, given the asymptomatic nature of lung cancer development in early stages. Unfortunately, the overall 5-year survival rate for advanced lung cancer is very low (< 3% in smokers and 8% in never smokers) [2, 3].

Smoking is the primary cause of lung cancer. Smoking cessation policies and interventions have substantially decreased smoking rates, contributing to the decline in lung cancer incidence in Northern American, European, and some Asian populations [4–8]. However, despite decreasing rates of lung cancer in smoking populations, lung cancer rates in people who have never smoked have remained unchanged, with the result that this population comprise an increasing proportion of lung cancer cases overall [9, 10]. Approximately, 10 to 25% of lung cancer cases occur in never-smokers in Western populations, while in Asian countries, it exceeds 50%, making it the fifth most common cancer in never-smokers. For example, in Canada and the UK, 5600 (15% of all lung cancer cases) and 6800 (14% of all lung cancer cases) of lung cancer cases are diagnosed in people who have never smoked each year, respectively [11, 12]. In China, 86.1% of lung cancers in females and 44.9% of lung cancers in males are diagnosed in never-smokers [13, 14]. The major predictors of lung cancer in people who have never smoked are outdoor air pollution (15% of all lung cancer deaths), second-hand smoking (5.8%), household air pollution (4%), radon (4%), and other exposures such as diesel exhaust, occupational exposures, arsenic, asbestos, and genetic susceptibility [15–23].

Early detection of lung cancer in people who have never smoked is an important public health priority. Detecting lung cancer in people who have never smoked at early stages can result in diagnosing asymptomatic patients when they are more likely to respond better to cancer treatment, increasing their chances of survival and reducing associated medical costs [24]. Several effective risk prediction models have been developed [25–30]. These are currently informing public health recommendations to identify high-risk individuals for lung cancer screening among smoking populations [31]. Currently, people who have never smoked are ineligible for lung cancer screening outside of some East Asian countries [32, 33]. With the increasing proportion of never-smokers among lung cancer cases, there is a pressing need to develop methods to identify high-risk individuals among the people who have never smoked and include them in lung cancer screening programs.

A recent narrative review identified four risk prediction models for lung cancer in people who have never smoked [33]. It was noted that these models included a few variables for predicting lung cancer, such as basic demographic characteristics, physical assessments, and cancer history, while failing to include the known predictors for lung cancer in people who have never smoked. Additionally, these models exhibited poor predictive accuracy [33–37]. Consequently, there are concerns about the generalizability of these models to other settings or populations. A preliminary literature search identified several other prediction models that were not included in the narrative review [38–40]. To date, there is no systematic review that compares and summarizes the evidence of existing prediction models for lung cancer in people who have never smoked while evaluating their predictive performance.

Although, to our knowledge, there is no published systematic review, our preliminary search revealed two ongoing systematic reviews [41, 42]. One of these reviews aims to synthesize evidence of existing externally validated risk prediction models only, regardless of smoking status [41]. The second review considers prediction models developed for both smoking and nonsmoking populations, not specifically focusing on people who have never smoked [42]. In contrast, our systematic review is intended to provide a comprehensive summary of the evidence of all existing risk prediction models for lung cancer in people who have never smoked, regardless of whether they have been externally validated or not. Our review will aim to address the following research questions:

- What models have been developed, validated, or updated to predict the future risk of lung cancer in people who have never smoked?
- How effectively do existing risk prediction models accurately predict or identify individuals who develop lung cancer in people who have never smoked?

## Methods

This systematic review protocol was developed in accordance with the Checklist for Critical Appraisal and Data Extraction for Systematic Reviews of Prediction Modelling Studies (CHARMS) and the Preferred Reporting Items for Systematic Review and Meta-Analysis Protocols 2015 statement (Table S1) [43]. The review protocol was registered on the PROSPERO website (protocol reference # CRD42023483824) [44].

### Eligibility criteria

#### Types of studies and index prediction models

This review will include models developed, validated, or updated using data sources from various types of randomized and non-randomized study designs, including cohort (retrospective or prospective), case-cohort, case–control, and cross-sectional studies that utilized existing registries (e.g., health administrative, hospital registries) to construct prediction models. We will include all model development studies, regardless of whether they performed external validation or not. Model development studies without external validation are defined as studies that aimed to predict the outcome through multivariable analysis (e.g., regression models, machine learning algorithms) and assess model predictive performance within a development dataset (i.e., internal validation). Model development studies with external validation are defined as studies that in addition to internal validation evaluated model performance using an external dataset, separate from the development dataset, such as different populations or settings.

We will also include external validation studies (i.e., studies that only externally validate existing models) and those aimed at updating existing prediction models for lung cancer in individuals who have never smoked. These updates may involve adding or replacing predictors, adjusting model coefficients, or simplifying the number of predictors. We will exclude studies reporting on the following: (1) models developed, externally validated, or updated in smoking populations or in mixed populations where it is impossible derive a model for individuals who have never smoked; (2) models developed, externally validated, or updated for the sole purpose of assessing predictive performance of a specific predictor (i.e., models designed to estimate adjusted prognostic effect of a factor); and (3) models developed for diagnostic purposes. We will also exclude publications that are not in English language.

#### Study population and setting

These are the general population or healthy adults (18 years and over), including individuals from certain risk groups (e.g., Asian populations, females), who have not been previously diagnosed with lung cancer and who have never smoked cigarettes or who have smoked less than 100 cigarettes in their lifetime, irrespective of whether they have vaped or consumed tobacco through other ways. There is no restriction on settings and locations where models were developed and/or externally validated or updated.

#### Outcomes

We will consider clinically or histologically diagnosed lung cancer cases and lung cancer-related deaths based on medical records, vital statistics, cancer registries, or self-reported medical history of lung cancer. All types of lung cancer will be included, such as non-small cell lung carcinoma (e.g., adenocarcinoma, squamous cell carcinoma, large cell (undifferentiated) carcinoma), small cell lung carcinoma, and other types of lung tumors (e.g., lung carcinoid tumor). For example, the following ICD-10 codes can be used to define the lung cancer cases or deaths: C34.0 (main bronchus), C34.1 (malignant neoplasm of upper lobe, bronchus, or lung), C34.2 (middle lobe, bronchus, or lung), C34.3 (lower lobe, bronchus, or lung), C34.9 (bronchus or lung, unspecified), and D02.2 (carcinoma in situ of bronchus and lung).

### Comprehensive search strategy

Electronic searches, unrestricted by language, will be conducted in MEDLINE (Ovid), Embase (Ovid), Web of Science Core Collection (Clarivate Analytics), and Scopus to retrieve relevant results from their inception to 1 November 2023 (initial search) to 31 January 2024 (updated search). Additionally, we will search for conference abstracts, theses and dissertations, and preprints in the Europe PMC and Open-Access Theses and Dissertation databases. The search strategy has been developed in consultation with an information specialist by combining free-text key words identified based on the following criteria — population (P), index (I), comparator (C), outcomes (O), and Medical Subject Heading terms. The search strategy has been built around the three main concepts according to the PICOTS format, which is a specific format to systematic reviews of prediction models [45]: population — nonsmokers, outcome — lung cancer, and index models — prediction models. The detailed search strategy can be found in Table S2.

The MEDLINE search strategy will be adapted for use in other databases considering their specific search algorithms, database indexing, and thesauri (e.g., controlled vocabulary search terms). The search strategy will be modified and updated to incorporate new terms if new relevant keywords are identified through the search. Relevant studies will also be searched by forward and backward citation chasing approaches. Non-peer-reviewed publications and conference proceedings will be considered eligible if they contain relevant information. In instances where conference proceedings alone are identified, attempts will be made to contact authors to obtain full study reports for a more comprehensive analysis. Including non-peer-reviewed publications and conference proceedings, we aim to ensure the comprehensiveness of the systematic review and potentially mitigate publication bias.

### Study selection

Two independent reviewers (A. I. and A. A.) will screen titles and abstracts of the retrieved records against eligibility criteria using the Covidence systematic review software (Veritas Health Innovation, Melbourne, Australia). After the preliminary screening, the full text of potentially relevant studies or those of indeterminate relevance will be obtained. The reviewers will then evaluate the full texts independently to select studies for inclusion in the review based on the established criteria. Any disagreements between the reviewers in screening and selecting studies will be resolved through discussion with a third reviewer until a consensus is reached. Study authors will be contacted if the reviewers are unable to determine the eligibility of a study based on incomplete or unobtainable information. Individual reasons for exclusion of each study at the full-text level will be recorded and made available as supplementary material in the completed review. The study inclusion and exclusion process will be displayed in a PRISMA flow diagram [46].

### Data extraction and management

Two independent reviewers (A. I. and A. A.) will extract data from the included studies using a standardized data extraction form. This form has been developed using the CHARMS checklist for data extraction for prognostic and diagnostic prediction model studies [45] and will be piloted in a subset of the included studies. Any disagreements between the reviewers regarding the extracted data will be resolved by consulting a third reviewer to achieve consensus. If necessary, we will make efforts to obtain missing data directly from study authors. If a study has developed multiple models, we will extract data from the model suggested by the study authors. In situations where the authors do not specify a recommended model, we will select the one demonstrating the highest accuracy (i.e., discrimination), lower risk of bias, and greater parsimony as the preferred model and document our decision. If a study validated a model on multiple populations or settings, we will extract data from all reported populations or settings. The following core data will be extracted:

#### Study-level characteristics

- Study design (e.g., model development, model validation, model update)
- Data source (e.g., cohort, case-cohort studies, randomized clinical trials, registries)
- Study dates (e.g., duration, start and completion dates)
- Sample size — Number of participants and number of outcomes and number of outcomes per the number of included candidate predictors
- Missing data — Number of participants with any missing value, number of participants with missing data for each predictor, and how missing data was handled
- Funding source and potential conflict of interest of the study authors

#### Participant characteristics

- Participant eligibility criteria and recruitment approaches (e.g., inclusion and exclusion criteria, settings, locations, sampling techniques)
- Description of participants (e.g., age, sex, ethnicity, other sociodemographic characteristics, behavioral and clinical characteristics (e.g., alcohol consumption, comorbidities))

#### Outcome characteristics

- Definition of and method to assess the outcome
- Type of outcome (e.g., whether lung cancer was combined with other outcomes — for example, lung cancer was combined with other respiratory and/or intrathoracic cancers)
- Duration of outcome follow-up

#### Details of candidate predictors

- Definition and measurement of candidate predictors
- When candidate predictors were measured

#### Model characteristics

- Modelling approach (e.g., logistic, survival regressions, machine learning techniques)
- Satisfying model assumptions
- Approach undertaken to select predictors for multivariable modelling (e.g., all or pre-selected candidate predictors, full model approach, backward or forward method, criteria-based selection — *p*-value, Akaike information criterion)
- Shrinkage of predictor weights or regression coefficients (e.g., no shrinkage, penalized estimation)
- Model calibration measures (calibration plot, calibration plot, Hosmer–Lemeshow test) and discrimination measures (C-statistic, D-statistic, log rank) with corresponding confidence intervals
- Model evaluation — model performance testing approach: Model development dataset (e.g., splitting, bootstrap, cross-validation) or external validation (e.g., different population, setting, temporal)

#### Study results, interpretation, and discussion

- Results from final and additional multivariable models (e.g., basic, simplified, extended), including predictor weights or regression coefficients, intercept, model performance measures with corresponding standard errors, and confidence intervals
- Strengths and limitations of the model
- Availability of the model equation or algorithm
- Adherence to the TRIPOD reporting checklist [47]

The participant and study-level characteristics will be valuable for assessing the applicability of the study results. Study-level characteristics are also crucial for determining if studies had a sufficient number of participants to construct a prediction model, whether missing data could have impacted the final model selection and results, and whether the timing of the studies was associated with technological advancements and other environmental changes that might have influenced the predictive performance of the model.

### Study quality assessment

To assess the methodological quality and applicability of the included studies, we will use the Prediction Model Risk-of-Bias Assessment Tool (PROBAST). This tool was specifically designed for assessing the risk of bias (ROB) and applicability of studies that develop, validate, or update prognostic prediction models [48]. PROBAST comprises four domains: participants (e.g., assessing ROB and applicability related to data sources and participant enrollment), predictors (e.g., evaluating ROB and applicability in defining and measuring predictors), outcome (e.g., evaluating ROB and applicability in defining the outcome), and analysis (e.g., assessing ROB related to selected statistical approaches and important statistical considerations). Two independent reviewers (A. I. and A. A.) will rank included studies regarding the ROB concerns in each of the four domains and applicability concerns for the first three domains, as “high,” “low,” or “unclear.” Disagreements will be resolved through discussion; if needed, a third reviewer will be consulted. Like the data extraction, when a study has developed multiple models, PROBAST will be applied to the model suggested by the study authors or selected by considering model accuracy and model parsimony. If a study has validated a model on multiple populations or settings, we will assess the model across all reported populations or settings.

### Data synthesis

#### Narrative synthesis

We will provide a narrative summary of study findings, including sociodemographic characteristics of the study population, study location, number of candidate and final predictors included in the model, predictor selection procedure, prediction model, internal and external (if appropriate) validation procedures, predictive accuracy of the model, model strengths and weaknesses, and other related information (number of events, missing data and handling missing data, follow-up, outcomes, study design, and other model performance measures). To facilitate model comparisons, all review findings will be tabulated. The narrative summary will also incorporate the findings from the assessment of ROB and applicability concerns for each domain and overall, which will be then presented in a table.

#### Meta-analysis

If at least two studies are found to have externally validated the same prediction model, we will combine model performance measures to evaluate the model’s average performance across diverse settings and populations, as well as its projected performance in a future setting. Given the expected heterogeneity among validation studies (e.g., case mix, design, settings), model performance measures will be synthesized using a random-effects approach. We will use restricted maximum likelihood estimation with the Hartung-Knapp-Sidik-Jonkman approach to calculate a 95% confidence interval for the mean model performance [49]. This approach is used to better account for the uncertainty around estimating between-study variability. In meta-analyses where only two studies are present, both Wald-type and Hartung-Knapp-Sidik-Jonkman approaches will be conducted. Quantitative synthesis of the model’s predictive performance will be conducted using the “metafor” and “metamisc” packages in the R statistical software version 4.2.2 (R Development Core Team, Vienna, Austria) [50, 51].

Model performance measures, such as discrimination and calibration estimates, will be summarized separately. Calibration assesses the model’s ability to accurately predict the risk of the outcome, measuring the agreement between the expected number of lung cases (based on model predictions) and the actual observed cases in a dataset. A well-calibrated model predicts the mean risk of the outcome in each group close to the actual number of individuals who developed the outcome. Calibration can be represented in various ways, such as a calibration plot, calibration slope, observed and expected number of events (O:E ratio), calibration table, or Hosmer–Lemeshow test. In contrast, discrimination refers to the model’s ability to distinguish or differentiate events from nonevents. Discrimination is usually measured as D-statistic, C-statistic, the area under the curve, or log-rank test [52]. Calibration and discrimination measures will be transformed to a logarithmical scale (logit C-statistic and log O:E ratio) to satisfy the normality assumption [53].

In cases where a sufficient number of studies (*n* ≥ 5) externally validated a common prediction model, we will calculate *I*^2^ statistic and ${\tau }^{2}$ — between-studies variance to assess and quantify heterogeneity of predictive performance measures (e.g., discrimination, calibration). If a sufficient number of studies are available, potential sources of heterogeneity will be explored using meta-regression analyses. Additionally, we will calculate prediction intervals to estimate the potential range model performance in a new validation study, when at least 10 studies are present.

If there is a sufficient number of studies, at least 10 studies, we will perform subgroup analysis stratifying by study location, study design (prospective vs retrospective and cross-sectional), ROB (e.g., “low” vs “high,” “low” vs “high” plus “unclear”), or source of data to investigate potential sources of heterogeneity. We will examine heterogeneity across subgroups using the chi-square test, where *p* < 0.1 will indicate statistically significant difference between subgroups. Models developed based on less than 50 cases who experienced the outcome during the follow-up period will be excluded from the meta-analysis, as such a low number of outcomes might not be sufficient for robust evaluation of model performance [45]. In the event that model performance measures such as calibration or discrimination are not reported, we will contact the study authors to obtain missing information. Models with missing model performance measures, even after attempted contact with the study authors, will not be included in the meta-analysis.

#### Sensitivity analysis

If there is a sufficient number of studies, we will perform sensitivity analysis among validation studies with low ROB, excluding studies assessed at unclear or high risk of bias. A similar analysis will be conducted excluding those studies that were assessed as of unclear or high concerns for the applicability. We will also test the robustness of the results by including the model performance measures derived from internal validation. Lastly, if the meta-analysis is feasible, we will include conference proceeding data, given sufficient data are reported.

#### Reporting deficiencies

To mitigate the potential for publication bias, we will perform a comprehensive systematic search including gray literature databases. We will also examine small study effects, including publication bias, if there is a sufficient number of studies (*n* ≥ 10) per a model, by visually inspecting asymmetry in a funnel plot and the Egger test for asymmetry [54].

### Reporting and dissemination

The review results will be reported according to the guidelines outlined in the Transparent Reporting of Multivariable Prediction Models for Individual Prognosis or Diagnosis: Checklist for Systematic Reviews and Meta-Analyses (TRIPOD-SRMA) and the Preferred Reporting Items for Systematic Reviews and Meta-Analysis (PRISMA) 2020 statement [46, 55]. Any variations from the established protocol will be documented and explained in the final report. Our findings will be shared through publication in peer-reviewed journals and presentation at scientific conferences.

The GRADE system was created to assist in guiding the interpretation of certainty across included study results in reviews of interventions [56]. Although the GRADE approach was adapted for rating the certainty of a body of evidence on prognosis in broad populations (overall prognosis) and prognostic factors [57, 58], there is no clear guidance on the application of the GRADE framework to prognostic prediction models. Some Cochrane systematic reviews on prognostic prediction models refrained from using GRADE and instead used the PROBAST risk-of-bias assessment tool to guide their judgement of the certainty of the body of evidence [59, 60]. We will also use the PROBAST tool in guiding our assessment of the certainty of the evidence.

## Discussion

With the increasing proportion of people who have never smoked among lung cancer cases, this review aims to enhance our understanding of the risk factors for lung cancer in the never-smoking populations. It will generate new insights to advance the early detection of lung cancer, potentially contributing to reducing lung cancer-related morbidity and mortality in people who have never smoked. The systematic review results will be useful for researchers planning to develop novel prediction models, as well as for clinical practitioners and policy makers seeking guidance for clinical decision-making and the formulation of future lung cancer screening strategies. Identifying and applying the most effective prediction model will facilitate personalized risk assessment for lung cancer, helping identify high-risk people who have never smoked and facilitating the optimal implementation of lung cancer screening programs in this population. Although the existing narrative review provides a good summary of the current literature, it comes with certain limitations when compared to the proposed review. This review will use more rigorous methods, systematically and comprehensively searching for risk prediction models, conducting comprehensive data extraction, and assessing risk of bias of each included study.

### Supplementary Information

**Additional file 1: Table S1.** PRISMA-P 2015 Checklist. **Table S2.** Primary literature search. Database: Ovid MEDLINE(R) and Epub Ahead of Print, InProcess, In-Data-Review & Other Non-Indexed Citations, Daily and Versions.

## Data Availability

Not applicable.

## References

[1] Sung H, Ferlay J, Siegel RL, Laversanne M, Soerjomataram I, Jemal A, Bray F (2021). Global cancer statistics 2020: GLOBOCAN estimates of incidence and mortality worldwide for 36 cancers in 185 countries. CA Cancer J Clin..

[2] SEER Cancer Stat Facts: Lung and Bronchus Cancer: National Cancer Institute. Bethesda, MD. Available from: https://seer.cancer.gov/statfacts/html/lungb.html. Accessed 5 Feb 2024.

[3] SEER*Explorer: an interactive website for SEER cancer statistics [Internet]: surveillance research program, National Cancer Institute; 2023 Apr 19. (updated: 2023 Jun 8). Accessed 26 Oct 2023. Available from: https://seer.cancer.gov/statistics-network/explorer/. Data source(s): SEER Incidence Data, November 2022 Submission (1975–2020), SEER 22 registries (excluding Illinois and Massachusetts). Expected Survival Life Tables by Socio-Economic Standards.

[4] Peto R, Darby S, Deo H, Silcocks P, Whitley E, Doll R (2000). Smoking, smoking cessation, and lung cancer in the UK since 1950: combination of national statistics with two case-control studies. BMJ.

[5] Zhang B, Ferrence R, Cohen J, Bondy S, Ashley MJ, Rehm J (2005). Smoking cessation and lung cancer mortality in a cohort of middle-aged Canadian women. Ann Epidemiol.

[6] Su Z, Jia X-H, Zhao F-H, Zhou Q-H, Fan Y-G, Qiao Y-L (2022). Effect of time since smoking cessation on lung cancer incidence: an occupational cohort with 27 follow-up years. Front Oncol.

[7] Khuder SA, Mutgi AB (2001). Effect of smoking cessation on major histologic types of lung cancer. Chest.

[8] Tse LA, Lin X, Li W, Qiu H, Chan CK, Wang F (2018). Smoking cessation sharply reduced lung cancer mortality in a historical cohort of 3185 Chinese silicotic workers from 1981 to 2014. Br J Cancer.

[9] Pelosof L, Ahn C, Gao A, Horn L, Madrigales A, Cox J (2017). Proportion of never-smoker non–small cell lung cancer patients at three diverse institutions. J Natl Cancer Inst..

[10] Cufari ME, Proli C, De Sousa P, Raubenheimer H, Al Sahaf M, Chavan H (2017). Increasing frequency of non-smoking lung cancer: presentation of patients with early disease to a tertiary institution in the UK. Eur J Cancer.

[11] Poirier AE, Ruan Y, Grevers X, Walter SD, Villeneuve PJ, Friedenreich CM (2019). Estimates of the current and future burden of cancer attributable to active and passive tobacco smoking in Canada. Prev Med.

[12] Cancer Statistics for the UK. Cancer Research UK. Available from: https://www.cancerresearchuk.org/health-professional/cancer-statistics/incidence/common-cancers-compared. Accessed 5 Feb 2024.

[13] Chen W, Xia C, Zheng R, Zhou M, Lin C, Zeng H (2019). Disparities by province, age, and sex in site-specific cancer burden attributable to 23 potentially modifiable risk factors in China: a comparative risk assessment. Lancet Glob Health.

[14] Wang J-B, Jiang Y, Wei W-Q, Yang G-H, Qiao Y-L, Boffetta P (2010). Estimation of cancer incidence and mortality attributable to smoking in China. Cancer Causes Control.

[15] Malhotra J, Malvezzi M, Negri E, La Vecchia C, Boffetta P (2016). Risk factors for lung cancer worldwide. Eur Respir J.

[16] Dubin S, Griffin D (2020). Lung cancer in non-smokers. Missouri medicine.

[17] Besaratinia A, Pfeifer GP (2008). Second-hand smoke and human lung cancer. Lancet Oncol.

[18] Hori M, Tanaka H, Wakai K, Sasazuki S, Katanoda K (2016). Secondhand smoke exposure and risk of lung cancer in Japan: a systematic review and meta-analysis of epidemiologic studies. Jpn J Clin Oncol.

[19] Huang F, Pan B, Wu J, Chen E, Chen L (2017). Relationship between exposure to PM2. 5 and lung cancer incidence and mortality: a meta-analysis. Oncotarget..

[20] Myers R, Brauer M, Dummer T, Atkar-Khattra S, Yee J, Melosky B (2021). High-ambient air pollution exposure among never smokers versus ever smokers with lung cancer. J Thorac Oncol.

[21] Silverman DT, Samanic CM, Lubin JH, Blair AE, Stewart PA, Vermeulen R (2012). The diesel exhaust in miners study: a nested case–control study of lung cancer and diesel exhaust. J Natl Cancer Inst.

[22] Hosgood HD, Wei H, Sapkota A, Choudhury I, Bruce N, Smith KR (2011). Household coal use and lung cancer: systematic review and meta-analysis of case–control studies, with an emphasis on geographic variation. Int J Epidemiol.

[23] Collaborators G, Ärnlöv J (2020). Global burden of 87 risk factors in 204 countries and territories, 1990–2019: a systematic analysis for the Global Burden of Disease Study 2019. Lancet.

[24] Whitaker K (2020). Earlier diagnosis: the importance of cancer symptoms. Lancet Oncol.

[25] Spitz MR, Hong WK, Amos CI, Wu X, Schabath MB, Dong Q (2007). A risk model for prediction of lung cancer. J Natl Cancer Inst.

[26] Tammemagi CM, Pinsky PF, Caporaso NE, Kvale PA, Hocking WG, Church TR (2011). Lung cancer risk prediction: prostate, lung, colorectal and ovarian cancer screening trial models and validation. J Natl Cancer Inst.

[27] Park S, Nam B-H, Yang H-R, Lee JA, Lim H, Han JT (2013). Individualized risk prediction model for lung cancer in Korean men. PLoS ONE.

[28] Kramer BS, Berg CD, Aberle DR, Prorok PC (2011). Lung cancer screening with low-dose helical CT: results from the National Lung Screening Trial (NLST).

[29] Infante M, Cavuto S, Lutman FR, Passera E, Chiarenza M, Chiesa G (2015). Long-term follow-up results of the DANTE trial, a randomized study of lung cancer screening with spiral computed tomography. Am J Respir Crit Care Med.

[30] Field JK, Vulkan D, Davies MP, Duffy SW, Gabe R (2021). Liverpool Lung Project lung cancer risk stratification model: calibration and prospective validation. Thorax.

[31] Field JK, Vulkan D, Davies MP, Baldwin DR, Brain KE, Devaraj A, et al. Lung cancer mortality reduction by LDCT screening: UKLS randomised trial results and international meta-analysis. Lancet Reg Health Europe. 2021;10:100179. 10.1016/j.lanepe.2021.100179 PMC8589726 34806061

[32] Wender R, Fontham ET, Barrera E, Colditz GA, Church TR, Ettinger DS (2013). American Cancer Society lung cancer screening guidelines. CA Cancer J Clin..

[33] Kerpel-Fronius A, Tammemägi M, Cavic M, Henschke C, Jiang L, Kazerooni E (2022). Screening for lung cancer in individuals who never smoked: an international association for the study of lung cancer early detection and screening committee report. J Thorac Oncol.

[34] Tammemaegi MC, Church TR, Hocking WG, Silvestri GA, Kvale PA, Riley TL (2014). Evaluation of the lung cancer risks at which to screen ever-and never-smokers: screening rules applied to the PLCO and NLST cohorts. PLoS Med.

[35] Wu X, Wen CP, Ye Y, Tsai M, Wen C, Roth JA (2016). Personalized risk assessment in never, light, and heavy smokers in a prospective cohort in Taiwan. Sci Rep.

[36] Warkentin MT, Lam S, Hung RJ (2019). Determinants of impaired lung function and lung cancer prediction among never-smokers in the UK Biobank cohort. EBioMedicine.

[37] Chien L-H, Chen C-H, Chen T-Y, Chang G-C, Tsai Y-H, Hsiao C-F (2020). Predicting lung cancer occurrence in never-smoking females in Asia: TNSF-SQ, a prediction model. Cancer Epidemiol Biomark Prev.

[38] Warkentin MT, Tammemägi MC, Espin-Garcia O, Budhathoki S, Liu G, Hung RJ (2022). Lung Cancer absolute risk models for mortality in an Asian population using the China Kadoorie Biobank. J Natl Cancer Inst..

[39] Liao W, Coupland C, Burchardt J, Baldwin D, initiative D, Gleeson F, Hippisley-Cox J. Predicting the future risk of lung cancer: development and validation of QCancer2 (10-year risk) lung model and evaluating the model performance of nine prediction models. medRxiv. 2022:2022.06. 04.22275868.

[40] Lan-Wei G. Lung cancer risk prediction nomogram in Chinese female non-smokers. Am Soc Clin Oncol. 2022;40(16_suppl):10530.

[41] Peltola L, Nilsson M, Torkki P, Jekunen A, Andersén H, Leskelä R-L, Nuutinen M. A systematic review and meta-analysis of lung cancer risk prediction models. PROSPERO. 2022;CRD42022321391. Available from: https://www.crd.york.ac.uk/prospero/display_record.php?ID=CRD42022321391. Accessed 5 Feb 2024. 10.2340/1651-226X.2025.42529 PMC12086449 40356086

[42] Juang YR, Seow WJ. Lung cancer risk prediction models in never-, formal- and current-smokers: a systematic review & meta-analysis. PROSPERO. 2022;CRD42022347087. Available from: https://www.crd.york.ac.uk/prospero/display_record.php?ID=CRD42022347087. Accessed 5 Feb 2024.

[43] Moher D, Shamseer L, Clarke M, Ghersi D, Liberati A, Petticrew M (2015). Preferred Reporting Items for Systematic Review and Meta-Analysis Protocols (PRISMA-P) 2015 statement. Syst Rev.

[44] Issanov A, Aravindakshan A, Puil L, Tammemägi M, Meza R, Lam S, Dummer T. Risk prediction models for lung cancer in people who have never smoked: a systematic review. PROSPERO. 2023;CRD420234838242023. Available from: https://www.crd.york.ac.uk/prospero/display_record.php?ID=CRD42023483824. Accessed 5 Feb 2024. 10.1186/s41512-024-00166-4 PMC10863273 38347647

[45] Moons KG, de Groot JA, Bouwmeester W, Vergouwe Y, Mallett S, Altman DG (2014). Critical appraisal and data extraction for systematic reviews of prediction modelling studies: the CHARMS checklist. PLoS Med.

[46] Page MJ, McKenzie JE, Bossuyt PM, Boutron I, Hoffmann TC, Mulrow CD (2021). The PRISMA 2020 statement: an updated guideline for reporting systematic reviews. Int J Surg.

[47] Collins GS, Reitsma JB, Altman DG, Moons KG (2015). Transparent reporting of a multivariable prediction model for individual prognosis or diagnosis (TRIPOD) the TRIPOD statement. Circulation.

[48] Wolff RF, Moons KG, Riley RD, Whiting PF, Westwood M, Collins GS (2019). PROBAST: a tool to assess the risk of bias and applicability of prediction model studies. Ann Intern Med.

[49] IntHout J, Ioannidis JP, Borm GF (2014). The Hartung-Knapp-Sidik-Jonkman method for random effects meta-analysis is straightforward and considerably outperforms the standard DerSimonian-Laird method. BMC Med Res Methodol.

[50] Debray T, de Jong V, Debray MT (2019). Package ‘metamisc’.

[51] Viechtbauer W, Viechtbauer MW. Package ‘metafor’. The Comprehensive R Archive Network Package ‘metafor’. 2015. Available from: http://cran.r-project.org/web/packages/metafor/metaphor.pdf. Accessed 5 Feb 2024.

[52] Alba AC, Agoritsas T, Walsh M, Hanna S, Iorio A, Devereaux PJ (2017). Discrimination and calibration of clinical prediction models: users’ guides to the medical literature. JAMA.

[53] Debray TPA, Damen JAAG, Snell KIE, Ensor J, Hooft L, Reitsma JB (2017). A guide to systematic review and meta-analysis of prediction model performance. BMJ.

[54] Sterne JA, Egger M. Regression methods to detect publication and other bias in meta‐analysis. Publication bias in meta‐analysis: prevention, assessment and adjustments. 2005:99–110.

[55] Snell KI, Levis B, Damen JA, Dhiman P, Debray TP, Hooft L, et al. Transparent reporting of multivariable prediction models for individual prognosis or diagnosis: checklist for systematic reviews and meta-analyses (TRIPOD-SRMA). BMJ. 2023;381:e073538. 10.1136/bmj-2022-073538 PMC10155050 37137496

[56] Goldet G, Howick J (2013). Understanding GRADE: an introduction. J Evid Based Med.

[57] Iorio A, Spencer FA, Falavigna M, Alba C, Lang E, Burnand B, et al. Use of GRADE for assessment of evidence about prognosis: rating confidence in estimates of event rates in broad categories of patients. BMJ. 2015;350:h870. 10.1136/bmj.h870 25775931

[58] Foroutan F, Guyatt G, Zuk V, Vandvik PO, Alba AC, Mustafa R (2020). GRADE guidelines 28: use of GRADE for the assessment of evidence about prognostic factors: rating certainty in identification of groups of patients with different absolute risks. J Clin Epidemiol.

[59] Moriarty AS, Meader N, Snell KI, Riley RD, Paton LW, Chew-Graham CA, et al. Prognostic models for predicting relapse or recurrence of major depressive disorder in adults. Cochrane Database Syst Rev. 2021(5):CD013491. 10.1002/14651858.CD013491.pub2 PMC8102018 33956992

[60] Kreuzberger N, Damen JA, Trivella M, Estcourt LJ, Aldin A, Umlauff L, et al. Prognostic models for newly‐diagnosed chronic lymphocytic leukaemia in adults: a systematic review and meta‐analysis. Cochrane Database Syst Rev. 2020(7):CD012022. 10.1002/14651858.CD012022.pub2 PMC8078230 32735048

